# JujubeNet: A high-precision lightweight jujube surface defect classification network with an attention mechanism

**DOI:** 10.3389/fpls.2022.1108437

**Published:** 2023-01-18

**Authors:** Lingjie Jiang, Baoxi Yuan, Wenyun Ma, Yuqian Wang

**Affiliations:** ^1^ School of Electronic Information, Xijing University, Xi’an, China; ^2^ Shaanxi Key Laboratory of Integrated and Intelligent Navigation, The 20th Research Institute of China Electronics Technology Group Corporation, Xi’an, China; ^3^ Xi’an Key Laboratory of High Precision Industrial Intelligent Vision Measurement Technology, Xijing University, Xi’an, China; ^4^ Humanities Teaching Department, Gansu University of Chinese Medicine, Dingxi, China; ^5^ Graduate Office, Xijing University, Xi’an, China

**Keywords:** agriculture 4.0, industry 4.0, artificial vision, CBAM, ConvNeXt

## Abstract

Surface Defect Detection (SDD) is a significant research content in Industry 4.0 field. In the real complex industrial environment, SDD is often faced with many challenges, such as small difference between defect imaging and background, low contrast, large variation of defect scale and diverse types, and large amount of noise in defect images. Jujubes are naturally growing plants, and the appearance of the same type of surface defect can vary greatly, so it is more difficult than industrial products produced according to the prescribed process. In this paper, a ConvNeXt-based high-precision lightweight classification network JujubeNet is presented to address the practical needs of Jujube Surface Defect (JSD) classification. In the proposed method, a Multi-branching module using Depthwise separable Convolution (MDC) is designed to extract more feature information through multi-branching and substantially reduces the number of parameters in the model by using depthwise separable convolutions. What’s more, in our proposed method, the Convolutional Block Attention Module (CBAM) is introduced to make the model concentrate on different classes of JSD features. The proposed JujubeNet is compared with other mainstream networks in the actual production environment. The experimental results show that the proposed JujubeNet can achieve 99.1% classification accuracy, which is significantly better than the current mainstream classification models. The FLOPS and parameters are only 30.7% and 30.6% of ConvNeXt-Tiny respectively, indicating that the model can quickly and effectively classify JSD and is of great practical value.

## 1 Introduction

SDD, also known as Automated Optical Inspection (AOI) or Automated Surface Inspection (ASI), is a significant research content in Industry 4.0 field (Penumuru et al., 2020). It is a technology that can acquire images by using machine vision equipment to determine whether there are defects in the acquired images. At present, surface defect equipment based on artificial vision has been widely used to replace manual visual inspection in various industrial fields, including automobile, household appliances, machinery manufacturing, semiconductor and electronics, chemical industry, medicine, aerospace, light industry, and other industries (Liu Y et al., 2020). For example: Ferguson et al. (2017) applied machine learning technology to the detection of surface defect on automobile parts. The GDXray Casting dataset adopted included 2,727 X-ray images, which were mainly from automobile parts, including aluminum wheels and steering knuckles. Tabernik et al. (2020) applied automated surface inspection technology to the detection of metal surface defects and published Kolektor SDD data set. Mayr et al. (2019) proposed a SDD method for solar panels. Huang et al. (2018) proposed a SDD method for magnetic tile surfaces. He et al. (2020) proposed An end-to-end steel SDD approach *via* fusing multiple hierarchical features. Gan et al. (2017) proposed hierarchical extractor-based visual rail surface inspection system. Yang et al. (2018) proposed automatic pixel-level crack detection and measurement using full convolutional network. Li et al. (2019) proposed detection algorithm for bridge cracks based on Deep Learning (DL). Tang et al. (2019) proposed an online Printed Circuit Board (PCB) defect detector on a new PCB defect dataset.

Compared with other computer vision tasks, SDD does not have a large and unified data set such as ImageNet (Deng et al., 2009), PASCAL-VOC (Everingham et al., 2010) and COCO (Lin et al., 2014). Defect detection mainly studies specific applications in different detection objects and scenarios. In the real complex industrial environment, SDD is often faced with many challenges, such as small difference between defect imaging and background, low contrast, large variation of defect scale and diverse types, large amount of noise in defect images, and even large amount of interference in the imaging of defects in natural environment. Therefore, it is faced with greater challenges.

The agriculture 4.0 model is an intelligent agricultural development model characterized by being intelligent and unmanned, and it is also a resource-integrated agricultural development model (Da Silveira et al., 2021). In the Agriculture 4.0 mode and the others, the difference between the agricultural management and service system is that, in the former mode, all the intelligent machinery and equipment, including their corresponding elements related to agriculture, such as agricultural production and circulation markets, are interconnected through the Internet of Things network. With the help of new internet technologies such as big data, cloud computing, and artificial intelligence, intelligent decisions on agricultural activities are made to improve the efficiency in resource utilization, labor productivity, and agricultural production. Lack of per capita resources, shortage of labor force, and urgent forms of environmental protection are scientific problems throughout the development of agricultural modernization (Zhai et al., 2020). Agriculture 4.0 is an in-depth development stage of agricultural modernization construction. Precise and intelligent agricultural production can be achieved with a higher level of intensity, precision, and coordination, and the three problems above can be fundamentally solved.

Jujube is a high-quality tonic native to China, rich in various vitamins, with high nutritional, edible, and medicinal values (Rashwan et al., 2020). Jujube has a history of being cultivated for more than 4,000 years. The quality is best for those with red color, thick, plump meat, small kernel, and sweet taste. The rapid increase in planting area of red jujube in China is in sharp contrast to the backward processing technology of post-harvest jujube. The jujube industry has boomed in recent years, with 3.3 million hectares under jujube cultivation and 7.46 million tons of production in China as of 2019. With the continuous improvement of living standards and the increasing popularity of food health knowledge, people’s demand for jujube products is also proliferating, and they have higher requirements for the quality of the fruit (Chen et al., 2017). However, during the natural growth, harvesting, and processing of jujube, defects such as deformation and rotten and cracked jujube fruit are often caused by pests, harsh environments, improper processing methods, and storage methods (Pham and Liou, 2020). If these defective products come onto the market mixed with the choicest jujube, it will seriously affect the jujube’s quality, sales, and prices, causing economic losses to jujube farmers and enterprises. Therefore, effective identification and classification of defective jujube is a primary means to ensure the quality of jujube products and has a significant economic value (GenG et al., 2019). Jujube appearance quality sorting technology is a crucial technology to improve jujube quality and product-added value in the process of jujube industrialization. Jujube sorting quality is also an important factor affecting the prices and markets of jujube. After the appearance and quality sorting, the market value of high-quality jujube can be increased, and the defective jujube can be made into new products through secondary deep processing, which is the direction and trend of the future revenue of the jujube industry. The traditional industrialization process of jujube requires a lot of manpower, and the deep processing of defective jujube can effectively save resources and reduce the pressure of environmental protection. Therefore, the application of artificial vision technology to the industrialization process of jujube production and the improvement in the level of automation can provide a beneficial reference for the combination of Agriculture 4.0 and Industry 4.0 (Araújo et al., 2021).

Machine vision technology has developed rapidly in recent years and has been gradually applied in the quality detection of agricultural products. However, at present, a large number of factories still use manual methods to classify JSD (Bhargava et al., 2022), which have obvious disadvantages such as low efficiency and high costs. Manual quality sorting is subject to significant fluctuations in human factors, and the phenomenon of wrong inspection and omission often occurs, which leads to the uneven overall quality of jujube commodities (Dong et al., 2022). Therefore, it is urgently necessary to introduce advanced technologies to innovate and replace the simple manual sorting methods to improve the quality of jujube products and achieve their automatic sorting.

Traditional SDD methods based on machine vision usually use conventional image processing algorithms or manually designed features and put into the classifier for classification. In general, the corresponding imaging scheme is usually designed according to the defect characteristics of the object surface to be inspected. A reasonable imaging scheme is helpful to obtain the image with uniform illumination and clearly reflect the surface defects of the objects. A common method is to select a light source based on the surface color of the object being inspected. For example, Jing et al. (2016) selected a composite white light source to image the various types of defects on the surface of colored fabrics. Another method is to select different imaging schemes according to the reflective properties of the object surfaces to be detected., mainly including bright field imaging, dark field imaging, and mixed imaging. As an example, Chen et al. (2016) designed two concentrically placed conic annular bright field light sources to illuminate the central and peripheral areas at the bottom of the metal can for SDD at the concave and convex bottom of the can. For the SDD algorithm of red jujube, Wu et al. (2016) used hyperspectral imaging techniques and machine vision algorithms based on Support Vector Machines (SVM) to achieve the quality classification.

In the real production environment, complex SDD often faces many challenges, such as low contrast, large variation of the defect scales, multiple defect types, noise, interference, etc. In this case, classical methods are often helpless, and it is difficult to obtain good detection results. The introduction of classical techniques did solve the automated sorting of red jujube to a certain extent. However, they require a high inspection environment and have problems such as low accuracy and poor real-time performance, and, therefore, are not conducive to large-scale promotion (Li et al., 2022). DL, a significant branch of machine learning, has made breakthroughs in recent years, especially with Convolutional Neural Networks (CNN), which have become widely used in various image recognition scenarios because of their powerful feature extraction and nonlinear representation capabilities, and some defect detection methods based on DL begin to have wide application in various industrial scenes. In 2014, Soukup and Huber-Mörk (2014) innovatively trained a CNN model to accurately classify cavity defects on the track surface by collecting photometric stereo images. The whole network consists of two convolutional layers, two pooling layers and the last fully connected layer. Ultimately, a recognition accuracy of 98.98% was achieved in the rail surface defect dataset. Park et al. (2016) designed a CNN-based SDD system for the automated detection of various defects, such as dirt, scratches, burrs, and wear, on the surface of parts in industrial production. This work shows that the method can achieve 98% classification accuracy on the validation dataset., and its detection speed is 5285 samples/min. Kyeong and Kim (2018) proposed a CNN framework to classify mixed-type defect patterns in Wafer Bin Map (WBM) of semiconductor industry. Deitsch et al. (2019) used the modified VGG19 network to identify defects in 300×300 resolution solar panel images, and the accuracy of the network reached 88.42%, which exceeds a variety of hand-designed features, including KAZE, Scale-Invariant Feature Transform (SIFT), Speeded Up Robust Feature (SURF), and the performance of the classifier exceeded the effect of the SVM method. Liang et al. (2019) proposed a method built on top of ShuffleNetV2 and achieved 99.88% classification accuracy on an in-line code inspection apparatus in the plastic container industry. The methods of directly using original images for SDD were widely used in many fields, such as welding defect classification (Zhang et al., 2019), lithium polymer battery bleb defect classification (Ma et al., 2019), PCB defect classification (Deng et al., 2018), etc. In addition, two-stage Faster R-CNN series and one-stage You Only Look Once (YOLO) series target detection networks are also used for SDD. For example: Tao et al. (2020) improved the two stage Faster R-CNN network for insulator defect location in drone power inspection, and Xue and Li (2018) realized shield tunnel lining defects detection based on the improved Faster R-CNN. Li et al. (2018) proposed a method based on MobileNet-SSD for the detection of sealing surface defects of filling production line containers. Liu et al. (2020) used MobileNet-SSD network to locate the high-speed rail catenary support components. Zhang et al. (2020) applied YOLOv3 to bridge surface defect location.

Several scholars have applied it to fruit sorting tasks and achieved successful results (Yin et al., 2022; Lin et al., 2022). Some researchers have also carried out relevant studies in the field of surface defect identification of jujube, which will be elaborated in the section “Related Work”. A CNN-based JSD classification algorithm named JujubeNet is proposed in this paper. The proposed algorithm is built on the basis of ConvNeXt (Liu et al., 2022) for the JSD detection. To solve the problem of SDD of jujube, the ConvNeXt model was improved as follows: firstly, by introducing a novel MDC module with a multi-branch structure instead of the original ConvNeXt module, the classification accuracy of the model is effectively improved, while the number of model parameters is reduced; Secondly, the CBAM is combined with ConvNeXt to make the model focus on different classes of jujube defects features (Woo et al., 2018). In the experiment of this paper, we compare JujubeNet with other mainstream algorithm models, and the results show that our JujubeNet has higher classification accuracy in the JSD classification scenario. The main contributions of this paper are as follows.

1. In the actual production environment, we collected 12000 images of jujube with 5 categories of defects (2000 images for each type of defective product) and 2000 images of good product, and created a dataset named ‘Jujube2000’, specifically for the classification study of surface defects of jujube. The jujube defect image dataset is released at https://pan.baidu.com/s/1mQZa_aoJ0uCitnSHta0UCg.
2. A MDC module with a multi-branch structure is designed, the CBAM is introduced to improve the ConvNeXt model, and finally, JujubeNet is proposed.
3. The effectiveness of the MDC module and CBAM Attention Mechanism (AM) was verified by ablation experiments, respectively.
4. The performance advantages of the proposed network in this paper are verified by comparing JujubeNet with the other mainstream networks.

The rest of this paper is structured as follows: Section 2 introduces the current DL-based algorithms for JSD. Section 3 introduces the ‘Jujube2000’ dataset and the improvement method proposed in this paper and presents JujubeNet. Section 4 compares and analyzes the experimental results of JujubeNet with the mainstream network on the ‘Jujube2000’ dataset. Section 5 summarizes the main work of this paper and indicates the directions for future research.

## 2 Related work

In recent years, DL methods represented by CNN have made significant breakthroughs in computer vision. The great success of AlexNet in the 2012 ImageNet competition also marks the beginning of the era of DL. Take image classification as an example, such as VGGNet (Simonyan and Zisserman, 2014), ResNet (He et al., 2016), and DenseNet (Huang et al., 2017), which have achieved satisfactory results in the traditional vision domain (Yang et al., 2022). Along with the development needs of smart agriculture and intelligent manufacturing, DL methods are increasingly introduced into various fields (Zhu et al., 2018). At present, scholars have gradually applied DL methods to fruit defect classification scenarios (Altalak et al., 2022). In this section, the current DL-based JSD classification methods will be discussed.

Geng et al. (2018) first applied a deep CNN with a two-branch structure to the defect classification task of jujube. In the first branch, the authors adopted a migration learning strategy to train the jujube dataset using SqueezeNet; in the second branch, the authors proposed a fusion module, which broadens the structure of the network and improves the classification accuracy of the model by contacting multiple feature maps. Finally, the classification model achieves an average accuracy of 99.3% on the self-built dataset of jujube. Sun et al. (2019) designed a CNN with low computational cost and high classification accuracy specifically for the real-time detection and classification of JSD. The model is based on DenseNet and simulates the visual mechanism of organisms by adding SE attention. In the authors’ self-built dataset of JSD, the model achieves classification accuracy comparable to mainstream networks with an accuracy of 91.9% with real-time availability. Wen et al. (2020) proposed a residual network-based method for classifying surface defects of jujube. First, the authors separated the G component from the RGB color map as the network’s input, improved the residual network’s activation and loss function, and then introduced the Dropout method to avoid overfitting. Finally, with the advantage of the residual module, the model achieved 96.1% classification accuracy on the self-built dataset of jujube. Guo et al. (2020) conducted a study on the impact of the jujube dataset on DL classification algorithms and used generative adversarial networks and rigid transformation to enhance the image data to solve the problem of an uneven sample of defective jujube. Experiments showed that the classification accuracy of the algorithm could be effectively improved, and based on the ResNet18 algorithm, the authors achieved a classification accuracy of 99.2%. Ju et al. (2022) proposed an improved ResNet for JSD classification. The authors first performed simulated data augmentation on small sample data to build a dataset containing five classes of JSD; secondly, the original ResNet was improved by embedding the SE module and applying Triplet loss and Center loss instead of Softmax loss; and finally, transfer learning was used to train the model. Their report shows that the classification accuracy of the algorithm can reach 94.2% in the authors’ self-built dataset of JSD. Yu (2022) proposed a multiple attention blending method for jujube grading. Using DenseNet121 as the backbone network, the authors obtained the final output by designing multiple attention mixing modules, specifically, constructing spatial attention, channel attention, and channel-space attention branches in the module and averaging the outputs of the three branches. The results show that the method can achieve a classification accuracy of 95.7%.

In summary, it is shown that the DL-based image classification algorithm is feasible for the JSD classification task. However, these studies are deficient in several aspects overall: Firstly, the dataset of JSD is not publicly available for academic research, which is detrimental to promoting the research on JSD. Secondly, the above studies are based on classical networks as the research basis of the model, which are not as good as the state-of-the-art networks in model accuracy and structural optimization design. In the Result section (**Table 1**), this paper demonstrates ConvNeXt’s superiority over other classical networks. In order to achieve more efficient industrial production, cutting-edge and practical techniques in academia should be applied to actual production. Finally, these studies lacked detailed analysis of the misidentified defects. A comprehensive and accurate analysis can be targeted to assist in the optimization of the model to improve the model’s classification accuracy and reduce misidentified. The purpose of this research is to design a lightweight and high-precision network for the classification of JSD. Considering the shortcomings of current research, this paper first collects and discloses the ‘Jujube2000’ dataset for academic research on the classification of JSD. Secondly, a novel MDC module is designed, and CBAM is introduced to improve the advanced ConvNeXt. Next, JujubeNet is proposed, specifically used for JSD classification. Then, the superiority of JujubeNet is demonstrated by extensive experiments. Finally, the direction of the model optimization in the following work was given by a detailed analysis of the misidentified case. In **Table 2**, this paper further summarizes the JSD classification methods mentioned above in terms of the algorithm used by the model, the composition of the data set, and the classification accuracy.

**Table 1:** Comparison of the performance for each model on the ‘Jujube2000’ test set.

Model	Accuracy	Precision	Recall	F1	FLOPS(G)	Params(M)
SqueezeNet*	75.7%	64.2%	75.7%	55.2%	**23.6**	**0.8**
VGG16	91.8%	93.4%	91.9%	91.9%	495.5	138.4
ResNet18*	93.4%	93.3%	93.4%	93.4%	58.2	11.2
ResNet34*	93.6%	93.7%	93.6%	93.6%	117.5	21.3
ResNet50*	93.4%	93.4%	93.5%	93.4%	131.5	23.5
DenseNet121*	93.8%	93.8%	93.8%	93.8%	91.7	7.0
Swin-Tiny	97.9%	97.9%	97.9%	97.8%	39.2	27.5
ConvNeXt-Tiny	98.6%	98.6%	98.6%	98.7%	142.6	27.8
JujubeNet (Ours)	**99.1%**	**99.1%**	**99.1%**	**99.1%**	43.7	8.5

The base algorithms used in each paper in **Table 1** are labeled using *, and bold data is the best result.

**Table 2:** Experimental results of algorithms in their own datasets in the relevant literature.

Reference	Basic Algorithm	Dataset Composition	Defect Category	Accuracy
Geng et al. (2018)	SqueezeNet	16000 for training 4000 for verification	4 Classes: Plump, Wizened, Cracked, Defective.	99.3%
Sun et al. (2019)	DenseNet	20100 for training 2355 for verification 1280 for testing	4 Classes: Invalid, Rotten, Wizened, Normal.	91.9%
Wen et al. (2020)	ResNet34	2800 for training 1120 for verification	3 Classes: Normal, Rotten, Cracked.	96.1%
Guo et al. (2021)	ResNet18	25129 for training 8424 for verification 7917 for testing	7 Classes: Black spot, Yellow skin, Cracked, Peeling, Wrinkled, Overlapping, Normal.	99.2%
Ju et al. (2022)	ResNet50	9478 for all	5 Classes: Health, Rotted, Split, Peeling, Russet.	94.2%
Yu et al. (2022)	DenseNet121	20929 for training 8969 for verification	5 Classes: Cracked, Split, Insect pest, Black spot, Normal.	95.7%

Since the dataset used in the relevant literature has not been publicly downloaded, it cannot be directly compared with the experimental results in the relevant papers. Therefore, this paper conducted a comparative experiment with the underlying networks in the related papers on the ‘Jujube2000’ dataset. The results show that the test accuracy of the base algorithms in the relevant papers on the ‘Jujube2000’ dataset is generally lower than that on their own datasets, which also reflects the fact that the ‘Jujube2000’ data set is more challenging and difficult to classify. Based on the ConvNeXt model, this paper achieved an improvement in model accuracy while significantly reducing FLOPS and the number of parameters. The experimental results show that our JujubeNet has a significant advantage in terms of prediction accuracy and parameter computation: the prediction accuracy reaches 99.1% on the ‘Jujube2000’ data set, and the number of parameters is only 8.5M. Please see the Result section of this paper for detailed results.

## 3 Materials and methods

### 3.1 Image shooting and processing

**Figure 1:**
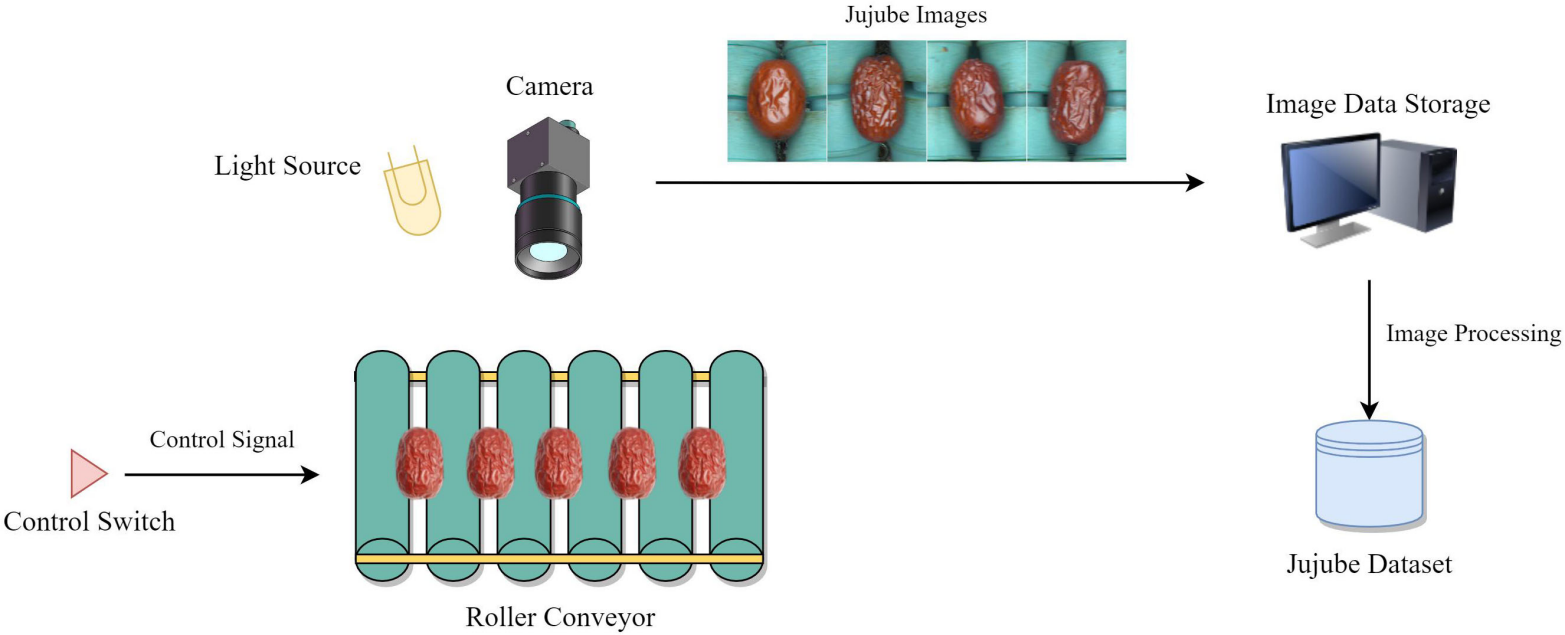
Workflow diagram of ‘Jujube2000’ data set collection platform.

Up to now, as the relevant literature has not disclosed the download links of their data sets, this paper has built a jujube image acquisition platform in the actual production scene, which is specially used for the collection of JSD images. The workflow of the acquisition platform is shown in **Figure 1**. The acquisition equipment mainly consists of LED light source, CCD industrial camera (MER-500-14U3C), roller conveyor, motor switch, and PC (CPU: AMD Ryzen 7 4800H 2.90GHz, RAM:16GB, SSD:500G).

**Figure 2:**
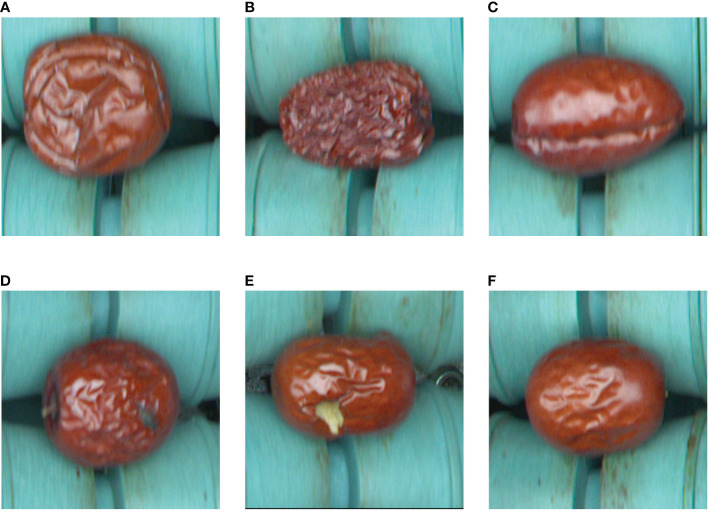
Examples of six kinds of defect samples: **(A)** deformed; **(B)** wrinkled; **(C)** cracked; **(D)** moldy; **(E)** bird-pecked; **(F)** normal. **(A-E)** are jujubes with surface defects, and **(F)** is one of high quality.

When the motor switch is activated, the roller conveyor drives the jujubes forward at a uniform speed, and at this time, the PC controls the camera to take and save the image. The camera can take a large image containing multiple jujubes at a time. Then, according to the gap between the rollers of the roller conveyor, the large image is divided into sub-images containing only one jujube. These sub-images are saved to the hard disk in png format. In order to obtain sufficient training images and guarantee the balance of the data set, 12000 images were selected from a large number of Jujube images to form the ‘Jujube2000’ data set, which contains 5 kinds of surface defects (2000 images for each defect) and 2000 high quality jujube images. The ‘Jujube2000’ dataset contains six categories: deformed, wrinkled, cracked, moldy, bird-pecked, and normal jujubes. The data set is divided into training, test and verification sets in a ratio of 7:2:1. Some sample images are shown in **Figure 2**.

During model training, multiple images need to be combined into a batch to feed the model (the batch size depends on the memory of the GPU), and the images must be of the same size in one batch, which makes the images need to be normalized before the model training. In a large dataset, there will inevitably be images of various sizes. Therefore, setting the height and width of images to be the same is an optimal solution considering the speed and accuracy of the model training (disadvantage: resize may lead to deformation of the object and thus affect the accuracy of the model). Numerous researches (He et al., 2016; Liu et al., 2022) have shown that using an input image size of 224 × 224 is the most balanced choice after considering the model accuracy and computational effort. For example, the default feature map size is 7 × 7 with a downsample multiplicity of 2. If the image size is adjusted to 112 × 112: the image information will be seriously lost. However, resizing the image to 448 × 448 will result in a significant increase in computing load. Therefore, in order to facilitate the model training, the jujube image size is normalized to 224×224 in this paper. Meanwhile, in order to improve the acquisition sample’s quality and increase the model’s generalization ability, this paper performs data enhancement on the images. The following are the approaches taken for the JSD classification scenario.

- Gaussian noise: Add noise to the image in line with the Gaussian distribution to simulate signal interference during image acquisition.
- Random cropping: On the one hand, The cropping of random regions on the image can have the effect of data enhancement; On the other hand, the stability of the model can be enhanced, and the model overfitting can be effectively prevented.
- Hybrid enhancement: Hybrid enhancement refers to the enhancement process of superimposing the contrast, brightness, and color of an image, and this operation contains a variety of image processing methods.
- Horizontal and vertical flip: Randomly flip the image horizontally or vertically without changing the original image information.

Finally, the ‘Jujube2000’ dataset contains 60,000 images after the image enhancement, of which 42,000 images are used for model training, 12,000 for model testing, and 6,000 for model validation.

### 3.2 Basic network selection

In order to achieve better classification results on the ‘Jujube2000’ dataset, this paper refers to the algorithms used by different authors in related works (Geng et al., 2018; Sun et al., 2019; Wen et al., 2020; Guo et al., 2020; Ju et al., 2021; Yu et al., 2022). However, the relevant literature did not disclose the code in their papers, and the data sets used were not publicly available for download. Therefore, this paper can neither directly identify the algorithms from the relevant literature for the ‘Jujube2000’ dataset for practical production nor directly compare the algorithms proposed in this paper with those in the relevant papers. For this reason, this paper evaluated the classification effect of the base models (SqueezeNet, ResNet18, ResNet34, ResNet50, DenseNet121) used in the related papers and the current mainstream base networks on the ‘Jujube2000’ dataset (detailed results of the experimental tests shown in Section 4.3). The evaluation results show that, firstly, SqueezeNet has the worst classification accuracy (only 75.7% accuracy in test dataset), which can not be directly applied in a real production environment; secondly, DenseNet121 has the slowest inference speed (DenseNet121: 40 images per second inference), which is not conducive to subsequent device deployment; Although VGG16 performed well in the training set, achieving a classification accuracy of 94.5%, it performed poorly in the test set, achieving only 91.8% classification accuracy, which also indicates the poor generalization ability of the network. Also, comparing the ResNet family, although they achieve similar accuracy on the test set, the difference in inference speed is more pronounced (ResNet18: 93.4% test accuracy, 251 images per second inference; ResNet34: 93.6% test accuracy, 168 images per second inference; ResNet50: 93.4% test accuracy, 115 images per second inference). Finally, ConvNeXt-Tiny achieved a classification accuracy of 97.5% on the training set. On the test set, ConvNeXt-Tiny has the best (98.6% test accuracy) generalization performance among all the mainstream networks participating in the comparison. Therefore, this paper further improved the network based on ConvNeXt-Tiny and got a better model named JujubeNet. After our improvements, the JujubeNet is a much lighter model than the ConvNeXt-Tiny, with less than 30.1% parameters and FLOPS, but 0.5% more accuracy, which makes it even more suitable for the processing scenario of industrial production of jujube.

### 3.3 ConvNeXt

**Figure 3:**
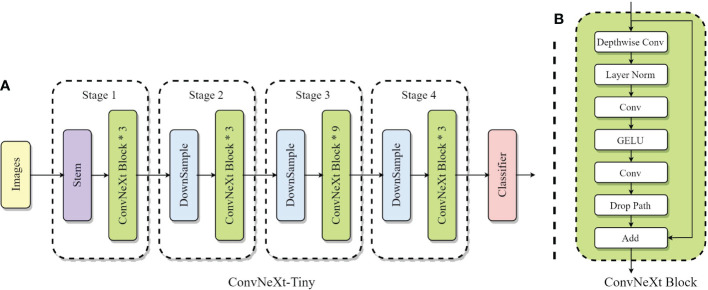
**(A)** ConvNeXt-Tiny overall network structure; **(B)** ConvNeXt Block structure.

ConvNeXt is a pure CNN proposed by FAIR in 2022 (Liu et al., 2022). It eliminates the need for tedious operations such as window shifting and relative position bias and offers better performance with less computation than the currently popular transformer network. The overall structure of ConvNeXt is based on the design of ResNet using residual blocks. It incorporates many advanced network design approaches to further improve the network’s overall performance. The detailed structure of ConvNeXt-Tiny and ConvNeXt Block are shown in **Figure 3**.

### 3.4. MDC block

**Figure 4:**
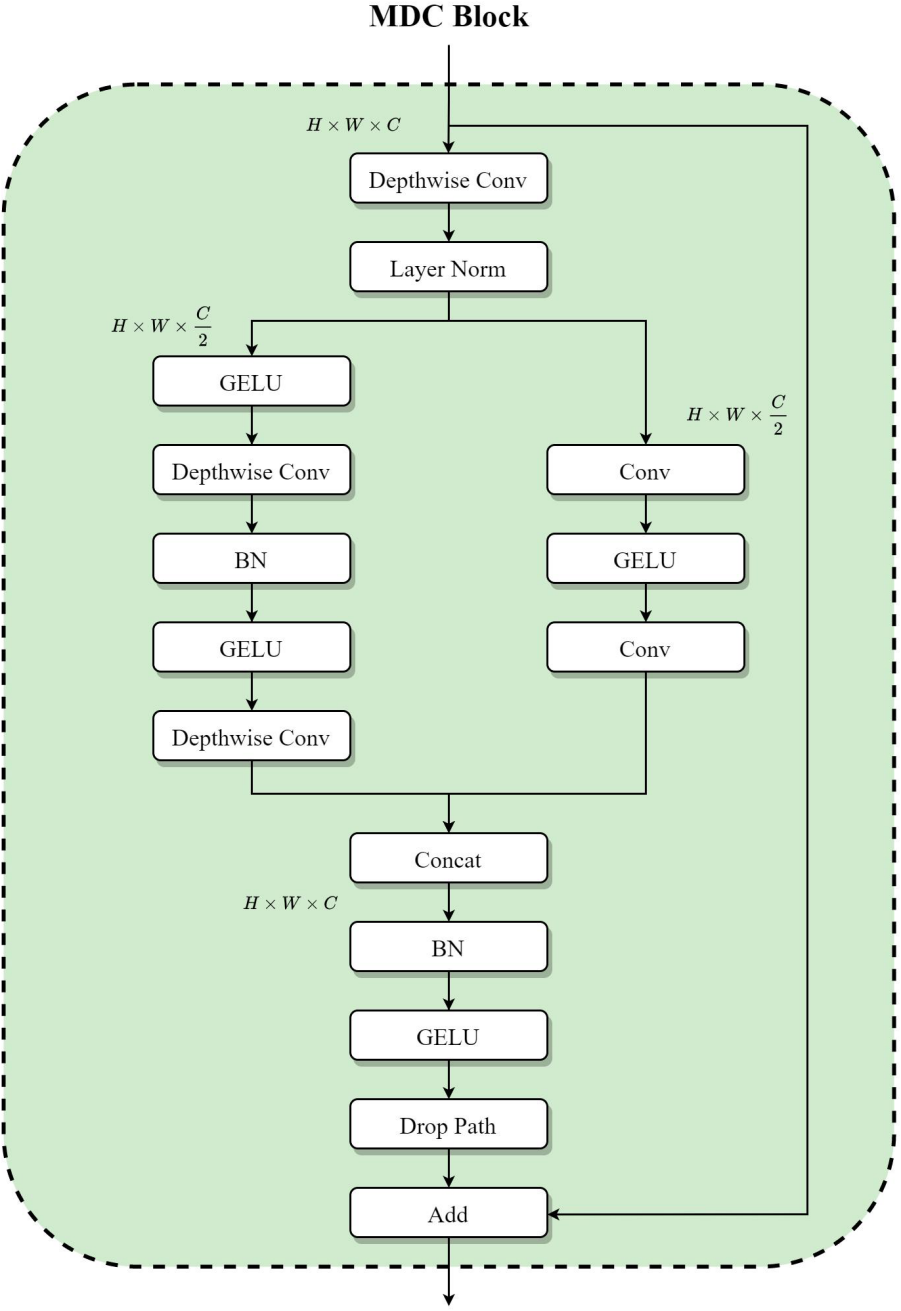
The structure of the MDC block.

Increasing cardinality is a more effective way of gaining accuracy than going deeper or wider. This idea was first proposed in ResNeXt (Xie et al., 2017). Drawing inspiration from it, this paper introduced a two-branch structure in the ConvNeXt Block. In one branch, the paper followed the original modular design; while in the other, it used two consecutive convolutional designs in reference to the Wide Residual Network (Zagoruyko and Komodakis, 2016). Of these two branches, each one’s channel dimension of each branch is half of that on the main branch. Meanwhile, this paper uses depthwise separable convolution in wide residuals instead of ordinary convolution to reduce the number of model parameters. Finally, after the two branches are dimensionally spliced, the information passing on the main branch is randomly discarded by Drop Path, which can effectively prevent the model from overfitting and improve the overall performance. The structure of the MDC block is shown in **Figure 4**.

### 3.5 CBAM

**Figure 5:**
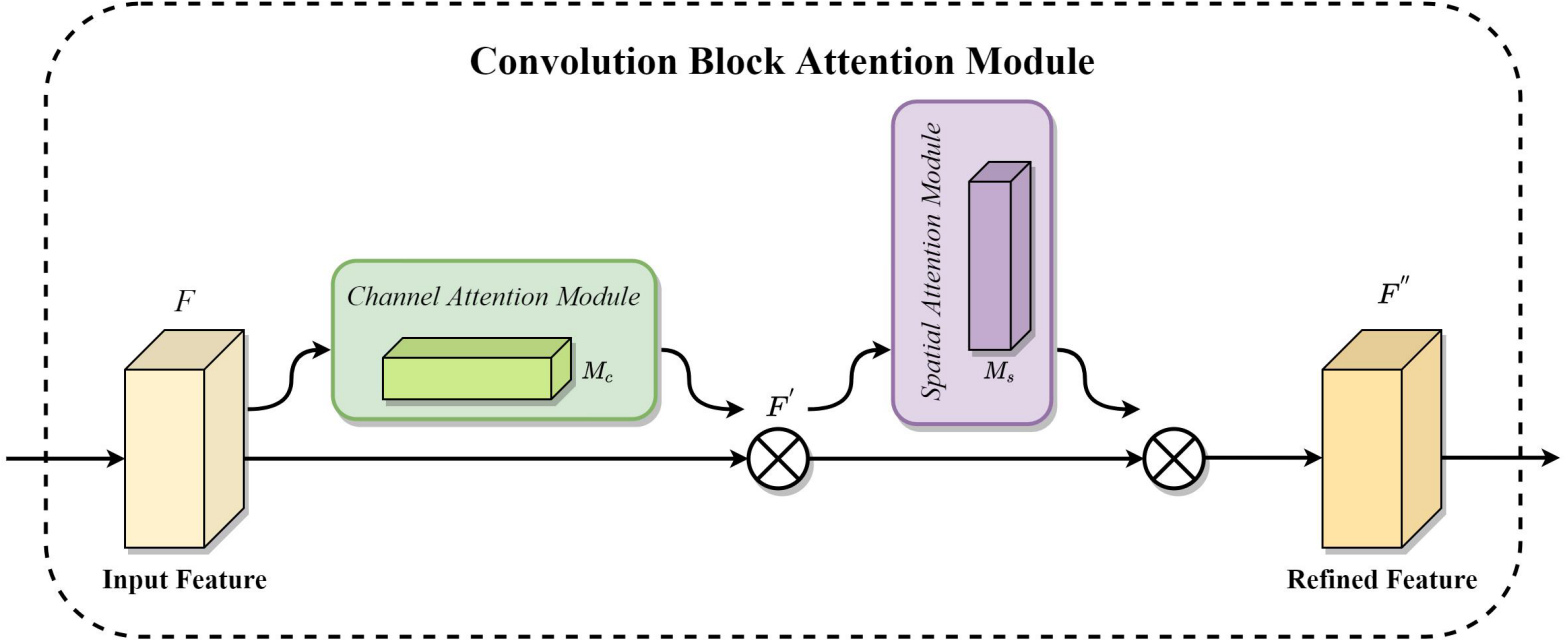
Convolution Block Attention Module.

In general, when CNN extracts features from the images, it will inevitably be disturbed by the background and noise, which can directly affect the classification effect of the networks. Such problems can be effectively overcome by introducing an AM, which enables the network to focus on the helpful feature information and suppress the useless noise and interference. This will improve the model’s classification accuracy. CBAM is a general and efficient AM proposed by Woo et al. in 2018, which perceives feature information in different dimensions through the channel attention module and focuses on location information in the feature map through the spatial attention module (Woo et al., 2018). Not only that but CBAM can also be easily integrated into CNN for end-to-end training (Zhong et al., 2022). The structure of CBAM is shown in **Figure 5**, which mainly consists of a channel attention module and a spatial attention module.

As shown in **Figure 5**, the feature map first passes through the channel attention module, which generates the corresponding channel attention map using the channel relationship between different features. Then, the input feature map is multiplied by the channel attention map. The output is again input to the spatial attention module, which generates the spatial attention map using the spatial position relationship between features. It multiplies the output of the channel attention module with the spatial attention map to obtain the final output feature map. The mathematical expressions of the above operations are shown in (1) and (2), where *F* (C×H×W) denotes the input feature map, *M_c_* (*F*) (C×1×1) is the one-dimensional channel attention map, *M_s_* (*F’*) (1×H×W) is the two-dimensional spatial attention map, ⊗ denotes the element multiplication operation, *F’* (C×H×W) is the output after the channel attention module, and *F’’* (C×H×W) is the final output of the CBAM.


(1) $$F^{\prime }=M_{c}\left(F\right)⊗F$$

(2) $$F^{\prime \prime }=M_{s}\left(F^{\prime }\right)⊗F^{\prime }$$

**Figure 6:**
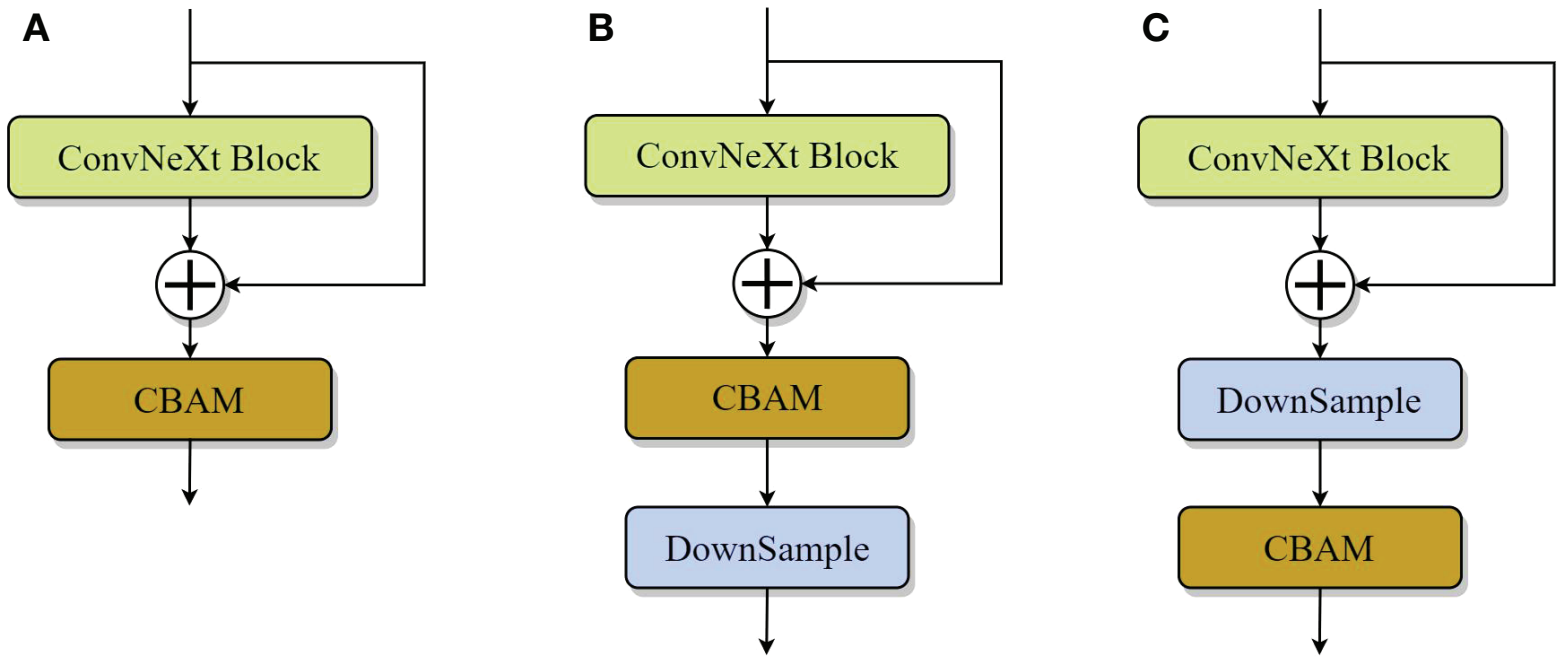
CBAM embedded position design.

This paper further explores the embedding position of CBAM in the model and designed the following three structures (Kim et al., 2021), as shown in **Figure 6**. Where (a) indicates the use of the CBAM after each ConvNeXt Block operation; (b) indicates that the model uses CBAM before each downsample; and (c) indicates that the network uses CBAM after each downsample, and according to the experiments in Section 4.1, (c) shows more excellent results, so our model will adopt the CBAM of (c) embedding position design.

### 3.6 JujubeNet

**Figure 7:**
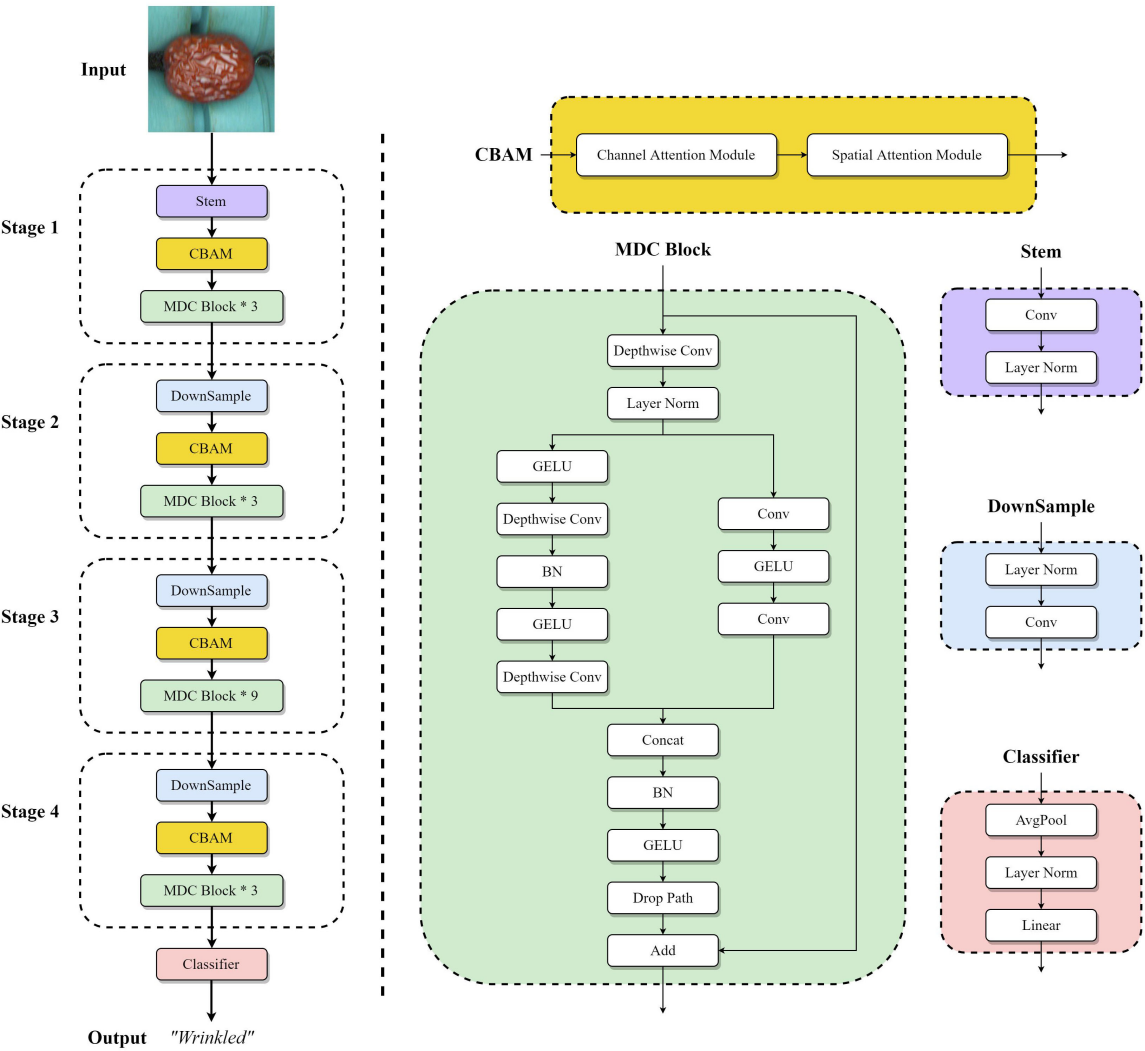
The network structure of the JujubeNet model. It consists of CBAMs (The details of the CBAM structure is shown in Section 3.5), MDC blocks, downsample modules, a stem module, and a classifier.

In this paper, a novel MDC module with multi-branch structure based on ConvNeXt Block is first designed, then introduced the CBAM AM into ConvNeXt, and finally proposed a high-precision lightweight classification network named JujubeNet, which is specifically designed for JSD classification. The experiments show that JujubeNet can perform the JSD classification excellently, and its overall network structure is shown in **Figure 7**.

### 3.7 Operating environment and parameter setup

All experiments were conducted on the same high-performance DL server (Central processing unit: Intel Xeon Silver 4210 CPU @2.20GHz; Graphics processing unit: NVIDIA GeForce RTX 2080 Ti 11GB; Memory: 128 GB; Deep learning framework: Python 3.8.10, Cuda 10.2, torch 1.8.1, torchvision 0.9.1; Operating system: Windows 10). In the experiments, uniform training parameters were set for all the models in this paper. The training image size is fixed at 224×224, the BatchSize set at 32, and the cross-entropy loss function and AdamW optimizer are applied in the model training. In the initial training phase of the models, this paper first performed a 2-epoch warm-up training, during which one-dimensional linear interpolation was employed to update the learning rate of each iteration. After the warm-up training, the learning rate is decayed using a cosine annealing function, where the initial learning rate is 0.0005, and the minimum learning rate is 0.00005. It is worth noting that we did not train the models using transfer learning in order to make a fairer comparison of the effects of network model modifications. Finally, each model was trained for 300 epochs.

### 3.8 Evaluate metrics

In DL methods, Accuracy, Recall, Precision, and F1-scores are significant metrics for evaluating the merits of classification models (Khasawneh et al., 2022). The accuracy rate is the percentage of the correct samples predicted by the model to the total number of samples; the recall rate is the percentage of the model correctly predicted as a positive sample to the total number of positive samples; the precision rate is the percentage of the number of positive samples predicted by the model that genuinely belongs to positive samples, and the F1-score is the best balance point that the model measures both the precision rate and the recall rate and achieves, and this value also reflects the overall performance of the model more comprehensively. Their specific formulae are shown below, where TP is the number of true positive samples, FP is the number of false positive samples, FN is the number of false negative samples, and TN is the number of true negative samples.


(3) $$Accuracy=\frac{TP+TN}{P+N}$$

(4) $$Recall=\frac{TP}{TP+FN}$$

(5) $$Precision=\frac{TP}{TP+FP}$$

(6) $$F1-Socre=\frac{2\times TP}{2\times TP+FP+FN}$$

## 4 Results

### 4.1 CBAM embedding position experiment

**Table 3** shows the detailed experimental results of the three CBAM embedding position schemes proposed in Section 3.5 of this paper. By observation, it can be found that as the state-of-the-art classification network, ConvNeXt exhibits strong classification performance, and the CBAM embedding position of the scheme (c) can further improve the model’s performance. Therefore, CBAM will be used after each downsample performed by the network.

**Table 3:** Performance comparison of different CBAM embedding position schemes.

Method	Accuracy	Precision	Recall	F1
ConvNeXt	98.6%	98.6%	98.6%	98.7%
ConvNeXt with (a)	98.7%	98.7%	98.7%	98.7%
ConvNeXt with (b)	98.5%	98.5%	98.5%	98.5%
ConvNeXt with (c)	98.8%	98.8%	98.8%	98.8%

### 4.2 Ablation experiments

To verify the effectiveness of the MDC module and CBAM in JujubeNet, this paper conducted ablation experiments on the test set, and the results are shown in **Table 4**.

**Table 4:** Comparison of ablation experiments on the test set.

MDC	CBAM	Accuracy	Precision	Recall	F1	FLOPS(G)	Params(M)
–	–	98.6%	98.6%	98.6%	98.7%	142.6	27.8
√	–	98.9%	98.9%	98.9%	98.9%	43.6	8.4
–	√	98.8%	98.8%	98.8%	98.8%	142.7	27.9
√	√	99.1%	99.1%	99.1%	99.1%	43.7	8.5

The data in the table shows that CBAM can effectively improve the model’s classification accuracy with almost no increase in the number of the model parameters, and the accuracy is improved by 0.3% compared with the original network. By using the MDC module, the model’s accuracy is also improved by 0.4%, and the FLOPS and the number of parameters are reduced by about 70%, making the model more efficient and lighter. Finally, the classification accuracy of JujubeNet reached 99.1% with the introduction of the two modules. The FLOPS and the number of parameters were only 43.7G and 8.5M, indicating that the MDC module and the CBAM AM can effectively enhance the model’s recognition of JSD and significantly reduce the number of the parameters.

### 4.3 Model comparison analysis

**Figure 8:**
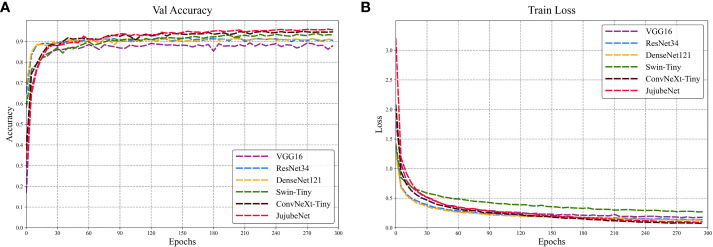
Training and validation of each model on the ‘Jujube2000’ dataset. **(A)** Trend of the validation accuracy curve of the model with the number of epochs. **(B)** Trend of the training loss curve of the model with the number of epochs. Colors denote corresponding models.

Under the experimental platform set in this paper, the performance of JujubeNet and the current mainstream classification models are compared on the ‘Jujube2000’ dataset. **Figure 8** shows the trend of validation accuracy and training loss with the number of epochs for each model. In **Figure 8A**, the horizontal axis indicates the number of rounds of model training and the vertical axis indicates the validation accuracy of the model. Similarly, in **Figure 8B**, the horizontal axis indicates the number of rounds of model training and the vertical axis indicates the training loss of the model.

**Figure 8A** shows that all the models start to converge at 30 epochs and slowly level off, reaching full convergence at 300 epochs. Among these models, JujubeNet achieved the highest validation accuracy, up to 96.0%, followed by ConvNeXt with 94.7% accuracy, and the VGG16 model had the worst validation accuracy of 88.3%. **Figure 8B** shows that the training loss curves of the CNN architecture models are relatively consistent, with the ConvNeXt model performing the best, reflecting its excellent fitting ability, while JujubeNet also shows its excellent ability. Swin-Tiny has the lowest convergence effect, which may be related to the fact that it is not trained using transfer learning because the transformer architecture model is more influenced by the number of training epochs and the dataset size.

All trained models were tested on the test set according to each performance metric, and the specific experimental results are shown in **Table 1**. The results show that JujubeNet has a significant advantage in terms of prediction accuracy and parameter computation. Based on the ConvNeXt model, this paper achieved a slight improvement in model accuracy while significantly reducing FLOPS and the number of parameters. Finally, JujubeNet achieves a prediction accuracy of 99.1%, and the number of parameters is only 8.5M. In addition, this paper also tested the classification effectiveness of the underlying networks in the related papers on the ‘Jujube2000’ dataset. The results show that the test accuracies of each network model on our dataset are generally lower than the test accuracies on their respective datasets, which also reflects the fact that ‘Jujube2000’ is more challenging and more difficult to classify.

### 4.4 Confusion matrix

**Figure 9:**
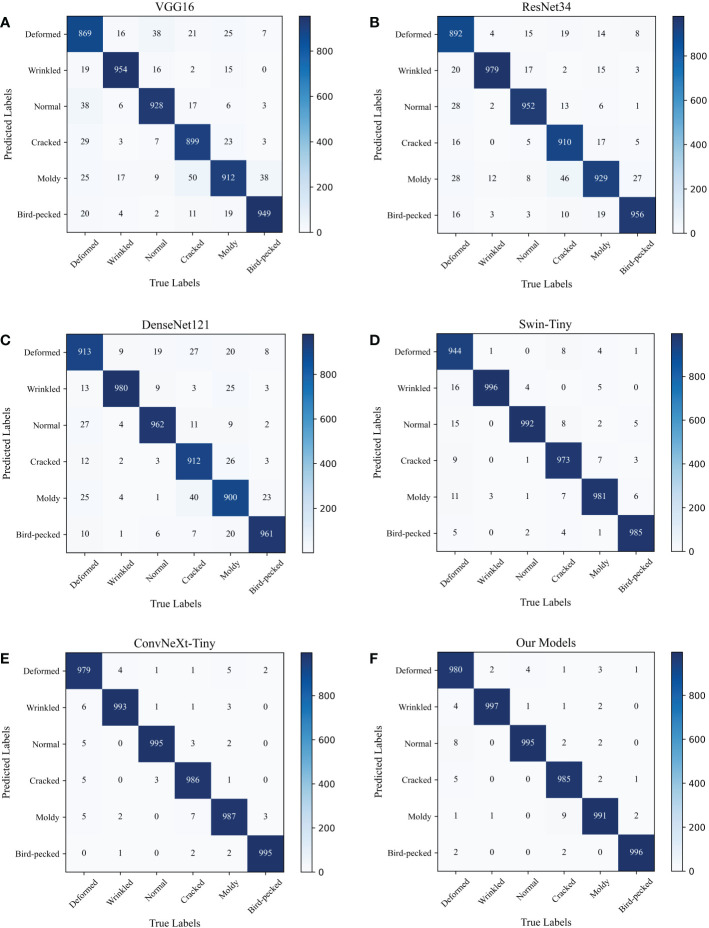
Confusion matrix for each model. **(A)** VGG16. **(B)** ResNet34. **(C)** DenseNet121. **(D)** Swin-Tiny. **(E)** ConvNeXt-Tiny. **(F)** The proposed JujubeNet.

The confusion matrix is a visual tool to evaluate the performance of a classification model, which describes the relationship between the prediction results of the model and the actual sample data. In addition, the confusion matrix can be used to more intuitively discern the strengths and weaknesses of the classification models and analyze their problems. As shown in **Figure 9**, this paper visualized the confusion matrix of each model in the previous subsection. The horizontal axis represents the true labels of the images, and the vertical axis represents the results predicted by the models. The diagonal part indicates that the predicted results of the models are the same as the true labels, and the remaining part indicates that the predicted results of the models do not match the true labels.

The analysis reveals that, firstly, the deformation defects of jujubes are the easiest to be misjudged because their defective characteristics are the least obvious. Second, crack defects are more likely to be misclassified as moldy, because the black spots of mold are more similar to the cracks of crack defects. Third, due to the variability of moldy defects, making this defect is more easily identified as other defect categories. Among the mainstream networks, Swin-Tiny and ConvNeXt-Tiny achieved better classification performance because they are more novel and have a more reasonable network structure compared with VGG16, ResNet34 and DenseNet121, among which, ConvNeXt-Tiny performs the best. Furthermore, the classification performance of ConvNeXt-Tiny can be further optimized by constructing novel MDC module and introducing the CBAM AM in JujubeNet.

**Figure 10:**
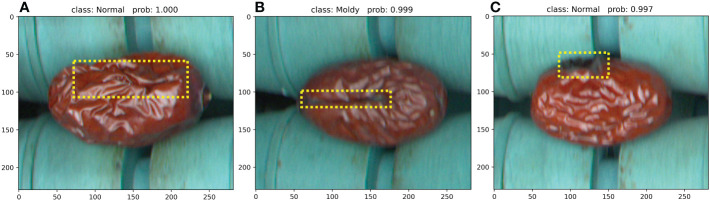
Examples of failure cases. The yellow dashed boxes indicate the defect areas that the model needs to focus. **(A)** A deformation defect was mistaken for a normal jujube. **(B)** A crack defect was mistaken for a moldy defect. **(C)** A moldy defect was mistaken for a normal jujube.

Jujubes are naturally growing plants, and defects of the same type may vary while defects of different types may differ less. Therefore, it is much more difficult than the defect detection of industrial products produced according to the specified process. Though JujubeNet shows state-of-the-art classification performance in many experiments, it has relatively more misidentified extruded, cracked, and moldy JSD in some cases. The specific details are shown in **Figure 10**. This paper tries to explore and analyze the causes of model discrimination errors for classifying anomalous images. **Figure 10A** shows that JujubeNet incorrectly identifies a deform defect as normal. The possible reason is that the shooting angle and lighting caused the defective sample to show more features of normal jujube, thus diluting the distortion. As shown in **Figure 10B**, even the human eye cannot accurately discern the specific defect of this jujube, as there appeared to be moldy in the cracked areas, which made it difficult for JujubeNet to make an accurate judgment. **Figure 10C** shows that a moldy defect was misidentified as normal. In this image, the moldy part is located on the edge of the jujube, causing the model to mistake it for a shaded area, leading to a classification error. Through a rational analysis of the failure cases, we will carry out targeted optimization on the proposed model to reduce misidentification in future work.

### 4.5 Visual interpretation of the model

**Figure 11:**
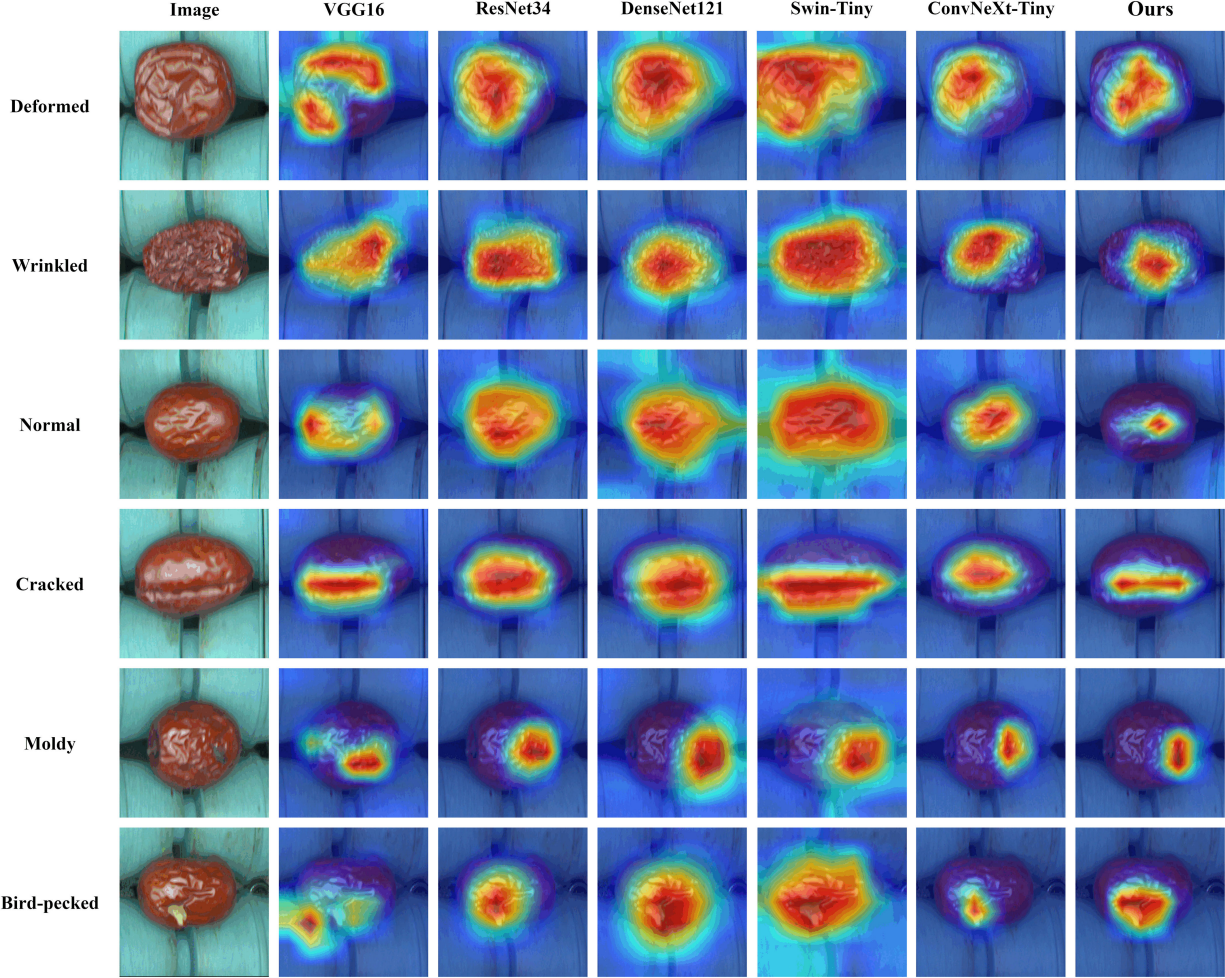
Comparison of heat maps generated by Grad-CAM for each model.

In order to further visualize and analyze the process of classifying surface defects of jujubes, this paper introduce Gradient-weighted Class Activation Mapping (Grad-CAM), which is a method of weighted summation of specific feature maps in the model by weight and outputs a heat map of the specified class (Li and Li, 2022). In the heat map, the higher the weight, the redder the region’s color, indicating that the image information of the region has a more significant influence on the model for category discrimination. Conversely, the smaller the weight, the bluer the region’s color, indicating that the image information of the region has less influence on the model for category discrimination. **Figure 11** shows the heat maps generated by each model using Grad-CAM in different categories of JSD.

The heat map gives us a clear view of the defect areas of interest to the model. The VGG16 model focuses on a more scattered region, so its classification is the least effective. ResNet34 and DenseNet121 models have more similar heat maps, which focus on a broader range of regions and have the problem of inaccurate focus on defective regions. Swin-Tiny naturally has the advantage of long-range information dependence because it is a transformer architecture model. Hence, it not only focuses on the most expansive area but also suffers from the problem of imprecise focus on defective regions. It can be found that ConvNeXt-Tiny can focus on the defective regions of jujube more precisely compared with the other networks, which makes the network achieve better classification results. In JujubeNet, by constructing the MDC module and introducing the CBAM AM, the position attention of the model to the defective regions is further optimized. Therefore, the classification performance is improved.

## 5 Conclusions

SDD is a significant research content in Industry 4.0 field. In the real complex industrial environment, SDD is often faced with many challenges, such as small difference between defect imaging and background, low contrast, large variation of defect scale and diverse types, large amount of noise in defect images. Jujubes are naturally growing plants, so the problem of SDD of jujube is also related to agriculture 4.0 field. Lack of per capita resources, shortage of labor force, and urgent forms of environmental protection are scientific problems throughout the development of agricultural modernization Agriculture 4.0 is an in-depth development stage of agricultural modernization construction. Precise and intelligent agricultural production can be achieved with a higher level of intensity, precision, and coordination, and the three problems above can be fundamentally solved. The rapid increase in planting area of red jujube is in sharp contrast to the backward processing technology of post-harvest jujube. The traditional industrialization process of jujube requires a lot of manpower, and the deep processing of defective jujube can effectively save resources and reduce the pressure of environmental protection. Therefore, the application of artificial vision technology to the industrialization process of jujube production and the improvement in the level of automation can provide a beneficial reference for the combination of Agriculture 4.0 and Industry 4.0.

In the actual production environment, we collected 12000 images and created a dataset named ‘Jujube2000’, which is specifically used for the classification study of surface defects of jujube. In this paper, a ConvNeXt-based high-precision lightweight classification network named JujubeNet is proposed for the defect classification of jujubes. Firstly, a MDC module with a multi-branch structure is designed, then, the CBAM is introduced to improve the ConvNeXt model, and finally, JujubeNet is proposed. In the ablation experiment phase, the effectiveness of the MDC module and CBAM AM was verified, respectively. In this paper, a comparative experiment is carried out on the ‘Jujube2000’ dataset with the underlying network in the relevant papers. By comparison, the performance advantage of JujubeNet is verified. The results show that our model exhibits better recognition accuracy and the FLOPS and number of parameters are much lower than the other models with the same performance, proving the effectiveness of the improved method proposed in this paper. In addition, some cases of classification errors were analyzed by confusion matrix and visualized. Future work will continue to study these difficult samples and further optimize the algorithm. The research results in this paper are not only applicable to the defect classification of jujubes but also can be extended to other defect classification scenarios.

## Data availability statement

The original contributions presented in the study are included in the article/**Supplementary Materials**. Further inquiries can be directed to the corresponding author.

## Author contributions

LJ and BY conceptualized of this study, selected the algorithm, designed and performed the experiment, analyzed the data, trained the algorithms, and wrote the original draft. WM collected data and revised the manuscript. YW and WM conceived the study and participated in its design. All authors contributed to this article and approved the submitted version.
