# Supplementary figures and images for: JujubeNet: A high-precision lightweight jujube surface defect classification network with an attention mechanism
Source: Front Plant Sci. 2023 Jan 18;13:1108437. doi: 10.3389/fpls.2022.1108437 (PMC9889997; doi:10.3389/fpls.2022.1108437)

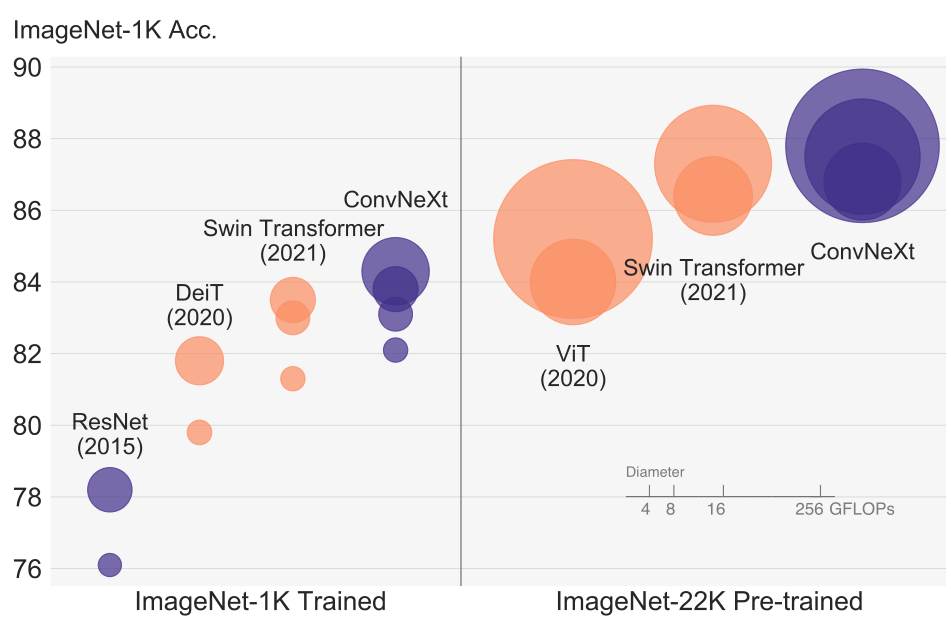

Supplement: Supplementary file 1 [file Image_1.png]

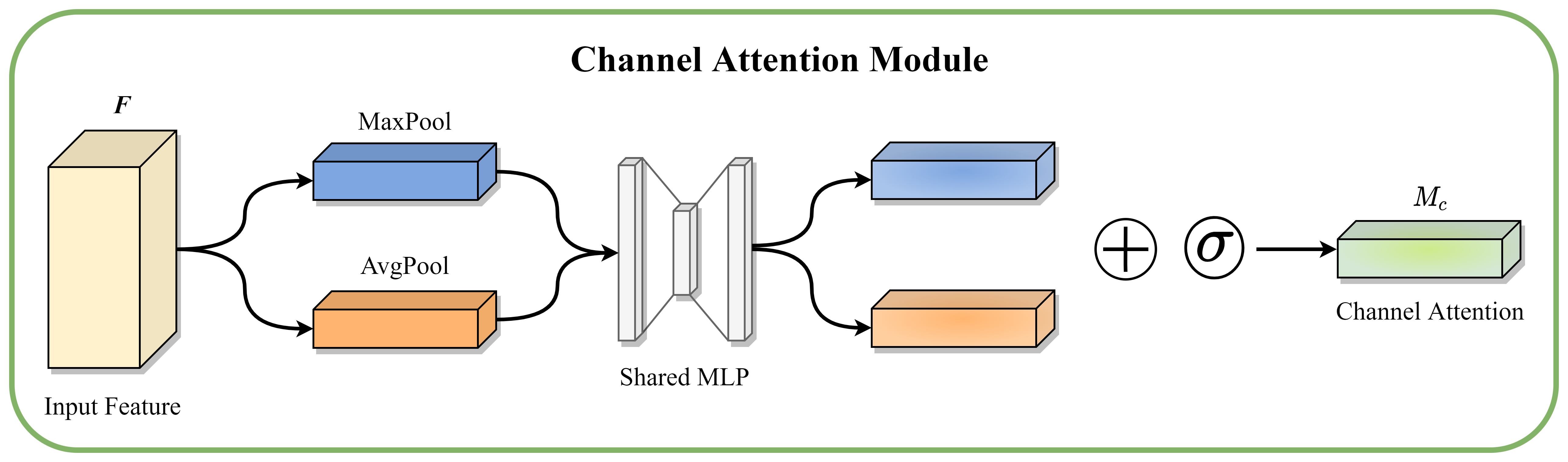

Supplement: Supplementary file 2 [file Image_2.png]

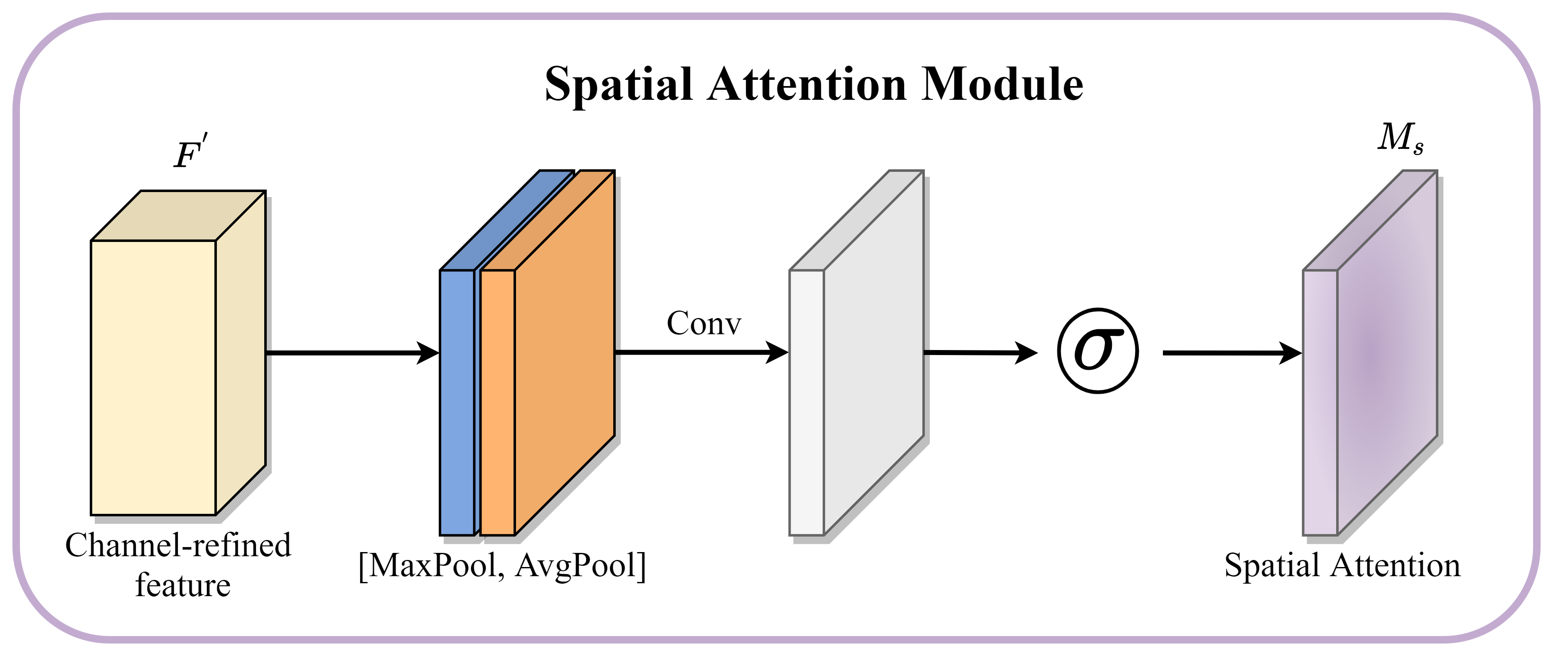

Supplement: Supplementary file 3 [file Image_3.png]
